# Supplementary material for: Application research of artificial intelligence software in the analysis of thyroid nodule ultrasound image characteristics
Source: PLoS One. 2025 Jun 2;20(6):e0323343. doi: 10.1371/journal.pone.0323343 (PMC12129332; doi:10.1371/journal.pone.0323343)
Supplement: S1 Files — This supplementary material provides detailed information on the methods, algorithms, and system architecture used in the development of the intelligent diagnosis system for thyroid nodule ultrasound imaging. (PDF) [file pone.0323343.s001.pdf]

**The research and development of the intelligent diagnosis system for thyroid nodule ultrasound imaging**

1. **Dataset Details:**

The intelligent diagnosis system for thyroid nodule ultrasound images was developed using a high-quality and diverse dataset consisting of 5,500 ultrasound video samples from 10 anatomical regions (thyroid, breast, cervical lymph nodes, axillary lymph nodes, carotid artery, liver, gallbladder, kidney, bladder, and ovary). Each region contributed 550 video samples, which were randomly divided into a development set (350 samples), a tuning set (100 samples), and a test set (100 samples) in a 350:100:100 ratio.

Specifically, for thyroid-related data, 550 ultrasound videos were divided into a development set (350 samples), a tuning set (100 samples), and a test set (100 samples). The training and validation processes utilized these subsets to ensure a robust evaluation of the AI model.

2. **Image Processing Algorithm:**

The image processing algorithm in this system employed the Ultrasound Super-Resolution Network (USR-Net). This network architecture comprises:147 layers with approximately 5 million parameters. Key features include skip connections and multi-scale feature fusion to enhance high-dimensional feature extraction efficiency. Split convolution was used to improve the ability to extract fine details and textures, while channel fusion was integrated to optimize the processing speed for dynamic ultrasound images. The image processing algorithm was trained using the development set, with low-quality images generated through degradation processing as inputs and the corresponding high-quality original images as targets. The optimization objective was to minimize the mean absolute error (MAE). After 500 epochs of training, the MAE stabilized, achieving convergence.

3. **Localization Analysis Algorithm:**

The localization analysis algorithm consisted of three components: Candidate Region Generation, Multi-Scale Similarity Network (MSS-Net) and Non-Maximum Suppression (NMS). Key characteristics of the MSS-Net included 130 layers with

approximately 4 million parameters, utilizing depthwise separable convolution to enhance computational efficiency. The NMS process employed an IOU threshold of 0.7 to select the final localization results, which significantly improved precision.

**4. Validation and Optimization:**

The dataset was divided into a development set for training and a tuning set for parameter optimization. Performance validation was conducted using the independent test set. The degradation process for training involved generating low-quality images as input and using the original high-quality images as the training target. The KMeans clustering algorithm was used in the candidate region generation phase to select the optimal window size (K=3). During the multi-scale similarity detection phase, the sigmoid loss function was optimized. The IOU threshold for NMS was determined based on ROC curve analysis, with 0.7 being the optimal value. To meet real-time dynamic analysis requirements, Compute Unified Device Architecture (CUDA) and TensorRT acceleration technologies were introduced, converting core algorithms into GPU kernel functions and leveraging mixed precision computation. This improved the processing speed by a factor of two.

**5. Performance Results:**

The image processing algorithm improved the Peak Signal-to-Noise Ratio (PSNR) from 21.16 (before processing) to 35.15 (after processing) and the Structural Similarity Index (SSIM) from 0.582 to 0.967. Statistical analysis (t-test) showed no significant differences in image quality across ultrasound machine brands, frequency ranges, and gain ranges after processing ($p > 0.05$), demonstrating effective image quality normalization. The localization analysis algorithm achieved a sensitivity of 95.04%, specificity of 94.17%, and accuracy of 94.74% on the test set. For thyroid-specific data, the sensitivity was 94.14%, specificity was 95.45%, and accuracy was 94.63%. The system's average processing time per frame was 18.72 milliseconds (maximum 25 milliseconds), meeting the real-time processing requirement of completing each frame analysis within 33 milliseconds.
