# Supplementary material for: Application research of artificial intelligence software in the analysis of thyroid nodule ultrasound image characteristics
Source: PLoS One. 2025 Jun 2;20(6):e0323343. doi: 10.1371/journal.pone.0323343 (PMC12129332; doi:10.1371/journal.pone.0323343)
Supplement: S1_Table — This table presents the proportion of agreement between two sonographers in evaluating characteristic indicators of thyroid nodules based on ultrasound imaging. (DOCX) [file pone.0323343.s002.docx]

Table 1 Proportion of agreement of characteristic indicators between two sonographers

| Features | Proportion of agreement |
| --- | --- |
| Echogenicity |  |
| Echotexture | 0.875 |
| Echogenicity level | 0.693 |
| With very hypoechoic | 0.646 |
| Aspect ratio | 0.886 |
| Border | 0.766 |
| Margin | 0.775 |
| Calcification | 0.717 |
