# Supplementary material for: Application research of artificial intelligence software in the analysis of thyroid nodule ultrasound image characteristics
Source: PLoS One. 2025 Jun 2;20(6):e0323343. doi: 10.1371/journal.pone.0323343 (PMC12129332; doi:10.1371/journal.pone.0323343)
Supplement: Attachment — Submitted filename: Peer Review Report - Thyroid.docx [file pone.0323343.s004.docx]

**Peer Review Report**

**Title:** Application Research of Artificial Intelligence Software in the Analysis of Thyroid Nodule Ultrasound Image Characteristics
**Authors:** Chen Xu, Zuxin Wang, Jun Zhou, Fan Hu, Ying Wang, Zhongqing Xu, Yong Cai

**Reviewer:** Luqman Abass
**Date:** 2/13/2025

**1. Originality of the Research**

The study presents original research evaluating the AI software SW-TH01/II for thyroid nodule ultrasound analysis. The results have not been published elsewhere to the best of my knowledge, and the study offers novel insights into AI's potential in thyroid diagnostics. The investigation of inter-method consistency and diagnostic efficiency adds valuable contributions to the literature.

**Recommendation:** Acceptable with minor revisions.

**2. Technical Quality of Experiments and Analyses**

The experiments adhere to high technical standards. The methods, including image selection, AI processing, and manual evaluation, are clearly described. Statistical analyses are appropriate, with kappa coefficients and agreement proportions well-documented. However, the description of AI model architecture and training processes lacks sufficient detail.

**Recommendations:**

- Provide additional information on the training dataset size, algorithm optimization, and performance validation metrics.
- Clarify the potential impact of image heterogeneity on AI performance.

**3. Validity and Support of Conclusions**

The conclusions align with the results presented, with SW-TH01/II showing high consistency and reduced analysis time. However, the statement regarding AI's applicability in primary care may be overly optimistic without further validation.

**Recommendations:**

- Reframe the conclusion to acknowledge the need for prospective clinical validation.
- Discuss potential limitations of the software's static image-based analysis.

**4. Clarity and Language Quality**

The manuscript is generally clear and well-structured. Nevertheless, grammatical errors and typographical mistakes are present, particularly in sections 2.5 and 3.1.

**Recommendations:**

- Conduct a thorough language review to correct minor errors.
- Revise technical terms for consistency (e.g., “dectects” should be “detects”).

**5. Ethical Standards and Research Integrity**

The study meets ethical standards with appropriate approval and consent procedures. The inclusion/exclusion criteria are clearly defined, and patient confidentiality is preserved through data anonymization.

**Recommendation:** Satisfactory.

**6. Adherence to Reporting Guidelines**

The manuscript largely adheres to reporting guidelines for diagnostic research. The data availability statement is present but lacks clarity regarding potential access for reproducibility.

**Overall Recommendation:** Minor Revisions

The manuscript demonstrates significant scientific merit, and its findings contribute to the growing application of AI in thyroid imaging. Addressing the above points will enhance the manuscript's clarity, completeness, and credibility.
