# Supplementary material for: Application research of artificial intelligence software in the analysis of thyroid nodule ultrasound image characteristics
Source: PLoS One. 2025 Jun 2;20(6):e0323343. doi: 10.1371/journal.pone.0323343 (PMC12129332; doi:10.1371/journal.pone.0323343)
Supplement: Attachment — Submitted filename: Rebuttal Letter.docx [file pone.0323343.s006.docx]

Rebuttal Letter

**Dear Editor and Reviewers,**

We sincerely appreciate your thoughtful comments and constructive suggestions on our manuscript (PONE-D-25-01972). Your detailed feedback has been invaluable in improving the quality and clarity of our work. We have carefully addressed each of your comments and revised the manuscript accordingly.

In this rebuttal letter, we have provided detailed replies to all the points raised, with our responses formatted in steel blue for clarity. Additionally, we have highlighted the major changes in the revised manuscript using lapis blue to ensure they are easily identifiable.

Thank you again for your time and effort in reviewing our paper. We hope that the revisions meet your expectations and address your concerns.

**Sincerely,**
Yong Cai

Point-to-point responses are made as follows:

Comments from Academic Editor

1. Please ensure that your manuscript meets PLOS ONE’s style requirements, including those for file naming.

Response:

Thank you for your feedback regarding the manuscript’s compliance with PLOS ONE’s style requirements. We have carefully reviewed the journal’s style guidelines and have adjusted the manuscript’s structure accordingly to ensure full compliance. If there are any additional areas that require further adjustments, please let us know, and we will address them promptly.

2. Please note that PLOS ONE has specific guidelines on code sharing for submissions in which author-generated code underpins the findings in the manuscript. In these cases, all author-generated code must be made available without restrictions upon publication of the work.

Response:

Thank you for pointing out PLOS ONE’s guidelines on code sharing. We confirm that no author-generated code was used in this study, as the findings are based entirely on the analysis of collected data and the application of existing statistical methods. Therefore, there is no code to share for this manuscript. If further clarification is needed, please let us know.

3. Please state what role the funders took in the study. If the funders had no role, please state: “The funders had no role in study design, data collection and analysis, decision to publish, or preparation of the manuscript.” If this statement is not correct you must amend it as needed. Please include this amended Role of Funder statement in your cover letter; we will change the online submission form on your behalf.

Response:

Thank you for your feedback regarding the Role of Funder statement. We have revised the financial disclosure statement in the manuscript to include a description of the funders’ contributions. Specifically, we have added the following sentence: “The funders contributed to the study design, data collection and analysis, preparation of the manuscript, and the decision to publish.”Additionally, we have included this information in the cover letter as part of the financial disclosure statement. This addition ensures that the role of the funders is clearly and transparently stated in accordance with PLOS ONE’s guidelines. Please let us know if further modifications are required.

4. We note that you have indicated that there are restrictions to data sharing for this study. For studies involving human research participant data or other sensitive data, we encourage authors to share de-identified or anonymized data. However, when data cannot be publicly shared for ethical reasons, we allow authors to make their data sets available upon request. Please update your Data Availability statement in the submission form accordingly.

Response:

Thank you for your feedback regarding the Data Availability Statement. We have updated the statement in the manuscript to reflect that the data set used for analysis in this study has been uploaded as Supporting Information files. The revised statement now reads as follows: “The data set used for analysis in this study has been uploaded as Supporting Information files.” This ensures compliance with PLOS ONE’s data sharing policies. Please let us know if further adjustments are required.

5. Please review your reference list to ensure that it is complete and correct. If you have cited papers that have been retracted, please include the rationale for doing so in the manuscript text, or remove these references and replace them with relevant current references. Any changes to the reference list should be mentioned in the rebuttal letter that accompanies your revised manuscript. If you need to cite a retracted article, indicate the article’s retracted status in the References list and also include a citation and full reference for the retraction notice.

Response:

Thank you for your feedback regarding the reference list. We have carefully reviewed all references cited in the manuscript to ensure that they are complete, correct, and up to date. We confirm that none of the cited papers have been retracted. If any retracted articles were to be identified in future revisions, we will make the necessary updates to the reference list and provide a rationale for their inclusion or replace them with relevant current references, as per the journal’s guidelines. Please let us know if there are any additional concerns regarding the references.

Comments from Reviewer 1

The manuscript demonstrates significant scientific merit, and its findings contribute to the growing application of AI in thyroid imaging. Addressing the above points will enhance the manuscript's clarity, completeness, and credibility.

1. Provide additional information on the training dataset size, algorithm optimization, and performance validation metrics.

Response:

Thank you for your valuable comments regarding the AI model training details. We have provided a detailed explanation addressing the training dataset size, model architecture, and validation methods below:

**(1)Dataset Details:**

The intelligent diagnosis system for thyroid nodule ultrasound images was developed using a high-quality and diverse dataset consisting of 5,500 ultrasound video samples from 10 anatomical regions (thyroid, breast, cervical lymph nodes, axillary lymph nodes, carotid artery, liver, gallbladder, kidney, bladder, and ovary). Each region contributed 550 video samples, which were randomly divided into a development set (350 samples), a tuning set (100 samples), and a test set (100 samples) in a 350:100:100 ratio.

Specifically, for thyroid-related data, 550 ultrasound videos were divided into a development set (350 samples), a tuning set (100 samples), and a test set (100 samples). The training and validation processes utilized these subsets to ensure a robust evaluation of the AI model.

**(2)Image Processing Algorithm:**

The image processing algorithm in this system employed the Ultrasound Super-Resolution Network (USR-Net). This network architecture comprises:147 layers with approximately 5 million parameters. Key features include skip connections and multi-scale feature fusion to enhance high-dimensional feature extraction efficiency. Split convolution was used to improve the ability to extract fine details and textures, while channel fusion was integrated to optimize the processing speed for dynamic ultrasound images. The image processing algorithm was trained using the development set, with low-quality images generated through degradation processing as inputs and the corresponding high-quality original images as targets. The optimization objective was to minimize the mean absolute error (MAE). After 500 epochs of training, the MAE stabilized, achieving convergence.

**(3)Localization Analysis Algorithm:**

The localization analysis algorithm consisted of three components: Candidate Region Generation, Multi-Scale Similarity Network (MSS-Net) and Non-Maximum Suppression (NMS). Key characteristics of the MSS-Net included 130 layers with approximately 4 million parameters, utilizing depthwise separable convolution to enhance computational efficiency. The NMS process employed an IOU threshold of 0.7 to select the final localization results, which significantly improved precision.

**(4)Validation and Optimization:**

The dataset was divided into a development set for training and a tuning set for parameter optimization. Performance validation was conducted using the independent test set. The degradation process for training involved generating low-quality images as input and using the original high-quality images as the training target. The KMeans clustering algorithm was used in the candidate region generation phase to select the optimal window size (K=3). During the multi-scale similarity detection phase, the sigmoid loss function was optimized. The IOU threshold for NMS was determined based on ROC curve analysis, with 0.7 being the optimal value. To meet real-time dynamic analysis requirements, Compute Unified Device Architecture (CUDA) and TensorRT acceleration technologies were introduced, converting core algorithms into GPU kernel functions and leveraging mixed precision computation. This improved the processing speed by a factor of two.

**(5)Performance Results:**

The image processing algorithm improved the Peak Signal-to-Noise Ratio (PSNR) from 21.16 (before processing) to 35.15 (after processing) and the Structural Similarity Index (SSIM) from 0.582 to 0.967. Statistical analysis (t-test) showed no significant differences in image quality across ultrasound machine brands, frequency ranges, and gain ranges after processing (p > 0.05), demonstrating effective image quality normalization. The localization analysis algorithm achieved a sensitivity of 95.04%, specificity of 94.17%, and accuracy of 94.74% on the test set. For thyroid-specific data, the sensitivity was 94.14%, specificity was 95.45%, and accuracy was 94.63%. The system’s average processing time per frame was 18.72 milliseconds (maximum 25 milliseconds), meeting the real-time processing requirement of completing each frame analysis within 33 milliseconds.

We hope this detailed explanation addresses your concerns. Due to space limitations, we have provided detailed descriptions of the dataset, model architecture, training and optimization process and performance validation as Supporting Information 1. In the revised manuscript, we have summarized these key points in the Materials and methods section.

2. Clarify the potential impact of image heterogeneity on AI performance.

Response:

Thank you for your insightful comment regarding the potential impact of image heterogeneity on the performance of the AI algorithm. We fully acknowledge that variations in image characteristics—caused by factors such as different machine models, imaging settings, and patient-specific conditions—can influence the performance of AI algorithms. In our study, we specifically addressed this challenge by incorporating diverse data sources during the algorithm development phase. The dataset included ultrasound images collected from ten different machine models (e.g., GE, Siemens, Mindray, Esaote, Hitachi, Toshiba, Philips, Samsung, Neusoft, VINNO) with varying frequency ranges and gain settings. These variations ensured the representation of a wide spectrum of imaging conditions, contributing to the algorithm's robustness and generalizability.

To mitigate the impact of heterogeneity, we utilized our proprietary Ultrasound Super-Resolution Network (USR-Net) to preprocess the images. This network effectively reduced noise, standardized image quality, and harmonized variations caused by different machine brands, frequency ranges, and gain settings. Postprocessing analysis, including PSNR and SSIM evaluations, showed that the processed images achieved consistent quality across different imaging conditions, with no statistically significant differences (p > 0.05). This preprocessing step minimized the influence of image heterogeneity on downstream tasks, such as anomaly detection and localization.

While our current approach successfully reduced the impact of image heterogeneity, we recognize that residual variations may still exist and could affect the AI's performance in subtle ways. Future studies will focus on more granular analyses of algorithm performance across specific sources of heterogeneity, including individual machine models and imaging settings, to further optimize the algorithm's adaptability to diverse clinical scenarios. We have expanded the discussion section of the manuscript to include a detailed analysis of the potential impact of image heterogeneity on AI performance.

3. Reframe the conclusion to acknowledge the need for prospective clinical validation.

Response:

We sincerely thank for the valuable feedback and suggestions. Based on your recommendation, we have revised the conclusion to acknowledge the need for further prospective clinical validation of the SW-TH01/II AI-assisted ultrasound tool. In the revised conclusion, we emphasize the importance of further prospective clinical validation “ Despite these promising findings, further prospective clinical validation is essential for confirming the software’s clinical applicability and ensuring its broader adoption in routine practice”.

4. Discuss potential limitations of the software's static image-based analysis.

Response:

Thank you for raising the important point regarding the limitations of relying on static images for analysis. We agree that static image-based analysis has inherent constraints compared to dynamic video analysis. In the revised manuscript, we have acknowledged this limitation and provided a justification for the use of static images in our study. Specifically, we stated: “SW-TH01/II relies solely on static ultrasound images for feature extraction and analysis. This design has inherent limitations compared to dynamic video analysis, which allows sonographers to observe nodules in real-time and across various angles. Dynamic imaging can provide additional details, such as vascular flow patterns and the interaction of nodules with surrounding tissues, which are not captured in static images. However, the decision to evaluate the AI model on static images was based on several practical considerations. First, static images are widely used in clinical practice as a standard format for documentation and retrospective analysis. They are commonly archived and reviewed for diagnostic purposes, making static image analysis highly relevant to real-world workflows. Second, static images offer greater standardization and reproducibility, minimizing variability caused by operator-dependent factors during image acquisition. This is particularly important for multi-center studies, where standardization is critical for ensuring consistent and comparable results across different datasets. Third, static image analysis simplifies the computational and data storage requirements, which can be a significant challenge when working with large volumes of dynamic video data. By focusing on static images, we were able to streamline data collection and analysis while maintaining consistency across the study. Despite these advantages, we acknowledge the limitations of relying solely on static images. Dynamic imaging undoubtedly provides richer diagnostic information, and future iterations of SW-TH01/II could incorporate dynamic video analysis to further enhance its diagnostic capabilities.”

5. Conduct a thorough language review to correct minor errors.

Response:

Thank you for pointing out the need for a thorough language review. We have carefully reviewed the entire manuscript to identify and correct minor language errors, improve clarity, and ensure consistency in terminology. We also paid special attention to grammatical accuracy, sentence structure, and overall readability.

6. Revise technical terms for consistency (e.g., “dectects” should be “detects”).

Response:

Thank you for noticing the inconsistency in technical terms and pointing out the specific example. In response to your comment, we have thoroughly reviewed the manuscript to ensure that all technical terms are used consistently and correctly throughout the text. Specifically, the typo “dectects” has been corrected to “detects,” along with a careful examination of other similar terms to avoid any remaining errors. We appreciate your attention to detail, which has helped us improve the overall quality and precision of the manuscript.

7. The manuscript largely adheres to reporting guidelines for diagnostic research. The data availability statement is present but lacks clarity regarding potential access for reproducibility.

Response:

Thank you for your feedback regarding the Data Availability Statement. We have updated the statement in the manuscript to reflect that the data set used for analysis in this study has been uploaded as Supporting Information files. The revised statement now reads as follows: “The data set used for analysis in this study has been uploaded as Supporting Information files.” This ensures compliance with PLOS ONE’s data sharing policies. Please let us know if further adjustments are required.

Comments from Reviewer 2

1. Data Availability: Ensure compliance with PLOS ONE’s open data policy by making an anonymized dataset available.

Response:

Thank you for your feedback regarding the Data Availability Statement. We have updated the statement in the manuscript to reflect that the data set used for analysis in this study has been uploaded as Supporting Information files. The revised statement now reads as follows: “The data set used for analysis in this study has been uploaded as Supporting Information files.” This ensures compliance with PLOS ONE’s data sharing policies. Please let us know if further adjustments are required.

2. Static vs. Dynamic Images: Justify why the AI model is evaluated only on static images.

Response:

Thank you for highlighting the distinction between static and dynamic image analysis and the potential limitations of relying solely on static images. We fully acknowledge that dynamic imaging provides additional diagnostic information, such as vascular flow patterns and the interaction of nodules with surrounding tissues, which are not captured in static images. However, as discussed in the revised manuscript, the decision to evaluate SW-TH01/II using static images was based on several practical considerations. Specifically: “SW-TH01/II relies solely on static ultrasound images for feature extraction and analysis. This design has inherent limitations compared to dynamic video analysis, which allows sonographers to observe nodules in real-time and across various angles. Dynamic imaging can provide additional details, such as vascular flow patterns and the interaction of nodules with surrounding tissues, which are not captured in static images. However, the decision to evaluate the AI model on static images was based on several practical considerations. First, static images are widely used in clinical practice as a standard format for documentation and retrospective analysis. They are commonly archived and reviewed for diagnostic purposes, making static image analysis highly relevant to real-world workflows. Second, static images offer greater standardization and reproducibility, minimizing variability caused by operator-dependent factors during image acquisition. This is particularly important for multi-center studies, where standardization is critical for ensuring consistent and comparable results across different datasets. Third, static image analysis simplifies the computational and data storage requirements, which can be a significant challenge when working with large volumes of dynamic video data. By focusing on static images, we were able to streamline data collection and analysis while maintaining consistency across the study. Despite these advantages, we acknowledge the limitations of relying solely on static images. Dynamic imaging undoubtedly provides richer diagnostic information, and future iterations of SW-TH01/II could incorporate dynamic video analysis to further enhance its diagnostic capabilities.”

In light of these considerations, static image analysis was chosen as a practical and standardized foundation for evaluating the AI model in this study. Nonetheless, we appreciate the valuable insights dynamic imaging can provide, and we recognize the limitations of relying solely on static images. As noted in the discussion, future iterations of SW-TH01/II could incorporate dynamic video analysis to capture additional diagnostic features and further enhance its clinical utility.We hope this addresses your concern and clarifies the rationale behind our methodology. Thank you for your constructive feedback, which has allowed us to refine the discussion and outline important directions for future research.

3. Patient Demographics: Include age, gender, and clinical factors in the dataset description.

Response:

Thank you for pointing out the absence of demographic and clinical variables in our study. We acknowledge that the lack of information such as age, gender, and other clinical factors is a limitation that may influence both nodule characteristics and diagnostic outcomes. In the revised limitation section, we have addressed this issue by stating: “Second, the study exclusively focused on the patients’ thyroid ultrasound images and did not collect their basic demographic or clinical information, leading to the absence of participant-related details in the results section. These details may influence nodule characteristics and diagnostic outcomes, and their inclusion could provide a more comprehensive evaluation of the software’s performance. Future studies should aim to incorporate such data to better understand how patient-specific variables impact both AI and sonographer interpretations.”

We fully agree that the inclusion of demographic and clinical data would enable a more comprehensive evaluation of the software’s performance and its applicability across diverse patient populations. In future research, we plan to design prospective studies that collect and analyze such data to better understand the interplay between patient-specific variables and diagnostic outcomes. This will allow us to further assess the robustness and clinical utility of the software in broader real-world settings. Thank you again for your valuable feedback, which has helped us refine our discussion and identify important directions for future work.

3. Subgroup Analysis: Analyze AI performance across different image sources and machines.

Response:

Thank you for your valuable suggestion regarding the analysis of performance across different image sources and machine types. In the algorithm development phase, we collected a diverse dataset of ultrasound images from ten different machine models and brands, including GE, Siemens, Mindray, Esaote, Hitachi, Toshiba, Philips, Samsung, Neusoft, and VINNO. This diversity was intended to ensure that the algorithm could generalize across a wide range of devices. The dataset includes images from 5500 cases, with an approximately even distribution of cases across the ten machine brands .However, in the formal study phase, while we explicitly specified the inclusion of data from these machine models, we did not record the exact association between individual images and their respective machines. This decision was made to focus on evaluating the algorithm’s overall performance across a heterogeneous dataset, independent of specific machine types, while ensuring that the collected data represented a broad spectrum of imaging conditions and device characteristics.

We acknowledge that analyzing performance across different machine types could provide additional insight into the algorithm’s generalizability and robustness. We have expanded the discussion section of the manuscript to include a detailed analysis of the potential impact of different image sources and machines on AI performance. In future studies, we plan to implement more granular tracking of the association between images and machine models, enabling a detailed performance evaluation across different devices. We appreciate your thoughtful feedback, which has highlighted an important area for future improvement.

5. Gold Standard Reliability: Report inter-observer agreement among sonographers.

Response:

Thank you for your valuable suggestion regarding the inter-observer agreement among sonographers. Following your comment, we conducted an analysis of inter-observer agreement between the two primary sonographers who independently interpreted the thyroid nodule ultrasound images. The results indicated that the agreement on certain imaging features was relatively low. We believe this observation highlights an important reality: inter-observer variability is an inherent challenge in the interpretation of ultrasound images. This variability reflects the subjective nature of image interpretation, even among trained professionals, and underscores the need for robust methodologies to address such inconsistencies.

To mitigate the impact of this variability, we carefully considered the reliability of the gold standard during the study design phase. Specifically, we incorporated a third, more experienced sonographer to adjudicate cases where the initial two sonographers disagreed. The third sonographer reviewed the case and facilitated a discussion to reach a consensus, which was then used as the final result. This adjudication process was implemented to address the potential limitations of inter-observer agreement and to enhance the accuracy and consistency of the gold standard used in our study.

Given these considerations, we believe the low agreement observed between the two primary sonographers, while important, should not detract from the validity of our study’s conclusions. Instead, it serves to highlight the inherent challenges of manual ultrasound interpretation and the importance of incorporating adjudication mechanisms in similar research. To maintain transparency, we have reported the inter-observer agreement results in the Supporting Information 1 for reference.

Table 1 Proportion of agreement of characteristic indicators between two sonographers

| Features | Proportion of agreement |
| --- | --- |
| Echogenicity |  |
| Echotexture | 0.875 |
| Echogenicity level | 0.693 |
| With very hypoechoic | 0.646 |
| Aspect ratio | 0.886 |
| Border | 0.766 |
| Margin | 0.775 |
| Calcification | 0.717 |

6. AI Model Training Details: Clarify training dataset size, architecture, and validation method.

Response:

Thank you for your valuable comments regarding the AI model training details. We have provided a detailed explanation addressing the training dataset size, model architecture, and validation methods below:

**(1)Dataset Details:**

The intelligent diagnosis system for thyroid nodule ultrasound images was developed using a high-quality and diverse dataset consisting of 5,500 ultrasound video samples from 10 anatomical regions (thyroid, breast, cervical lymph nodes, axillary lymph nodes, carotid artery, liver, gallbladder, kidney, bladder, and ovary). Each region contributed 550 video samples, which were randomly divided into a development set (350 samples), a tuning set (100 samples), and a test set (100 samples) in a 350:100:100 ratio.

Specifically, for thyroid-related data, 550 ultrasound videos were divided into a development set (350 samples), a tuning set (100 samples), and a test set (100 samples). The training and validation processes utilized these subsets to ensure a robust evaluation of the AI model.

**(2)Image Processing Algorithm:**

The image processing algorithm in this system employed the Ultrasound Super-Resolution Network (USR-Net). This network architecture comprises:147 layers with approximately 5 million parameters. Key features include skip connections and multi-scale feature fusion to enhance high-dimensional feature extraction efficiency. Split convolution was used to improve the ability to extract fine details and textures, while channel fusion was integrated to optimize the processing speed for dynamic ultrasound images. The image processing algorithm was trained using the development set, with low-quality images generated through degradation processing as inputs and the corresponding high-quality original images as targets. The optimization objective was to minimize the mean absolute error (MAE). After 500 epochs of training, the MAE stabilized, achieving convergence.

**(3)Localization Analysis Algorithm:**

The localization analysis algorithm consisted of three components: Candidate Region Generation, Multi-Scale Similarity Network (MSS-Net) and Non-Maximum Suppression (NMS). Key characteristics of the MSS-Net included 130 layers with approximately 4 million parameters, utilizing depthwise separable convolution to enhance computational efficiency. The NMS process employed an IOU threshold of 0.7 to select the final localization results, which significantly improved precision.

**(4)Validation and Optimization:**

The dataset was divided into a development set for training and a tuning set for parameter optimization. Performance validation was conducted using the independent test set. The degradation process for training involved generating low-quality images as input and using the original high-quality images as the training target. The KMeans clustering algorithm was used in the candidate region generation phase to select the optimal window size (K=3). During the multi-scale similarity detection phase, the sigmoid loss function was optimized. The IOU threshold for NMS was determined based on ROC curve analysis, with 0.7 being the optimal value. To meet real-time dynamic analysis requirements, Compute Unified Device Architecture (CUDA) and TensorRT acceleration technologies were introduced, converting core algorithms into GPU kernel functions and leveraging mixed precision computation. This improved the processing speed by a factor of two.

**(5)Performance Results:**

The image processing algorithm improved the Peak Signal-to-Noise Ratio (PSNR) from 21.16 (before processing) to 35.15 (after processing) and the Structural Similarity Index (SSIM) from 0.582 to 0.967. Statistical analysis (t-test) showed no significant differences in image quality across ultrasound machine brands, frequency ranges, and gain ranges after processing (p > 0.05), demonstrating effective image quality normalization. The localization analysis algorithm achieved a sensitivity of 95.04%, specificity of 94.17%, and accuracy of 94.74% on the test set. For thyroid-specific data, the sensitivity was 94.14%, specificity was 95.45%, and accuracy was 94.63%. The system’s average processing time per frame was 18.72 milliseconds (maximum 25 milliseconds), meeting the real-time processing requirement of completing each frame analysis within 33 milliseconds.

We hope this detailed explanation addresses your concerns. Due to space limitations, we have provided detailed descriptions of the dataset, model architecture, training and optimization process and performance validation as Supporting Information 1. In the revised manuscript, we have summarized these key points in the Materials and methods section.

7. Time vs. Accuracy Trade-off: Discuss whether AI’s faster speed affects its diagnostic quality.

Response:

Thank you for raising the important point regarding the trade-off between speed and diagnostic accuracy in AI-based analysis. To address your concern, we have expanded the discussion section to explicitly address this issue. Specifically, we have added the following content:“One of the key advantages of SW-TH01/II is its ability to analyze thyroid ultrasound images significantly faster than manual interpretation by sonographers. Its rapid analysis may raise questions about the trade-off between speed and diagnostic accuracy. It is important to emphasize that the primary role of SW-TH01/II is to serve as an auxiliary tool, assisting rather than replacing sonographers in diagnostic workflows. AI-based SW-TH01/II is designed to enhance standardization and reproducibility in feature analysis, reducing variability caused by human factors such as subjective judgment or fatigue. By automating the extraction and analysis of key image features, the software aims to reduce the workload of clinicians and improve overall efficiency, allowing them to focus on cases that require more detailed interpretation. Therefore, the software’s speed is not a substitute for accuracy but rather a means to streamline routine tasks and support clinicians in making more informed and timely decisions. Nevertheless, future studies should further evaluate the software’s performance in real-world clinical settings to confirm this balance and identify potential areas for optimization.”

We hope this addition clarifies our position and addresses your concern. Thank you for your constructive feedback, which has helped enhance the clarity and depth of our discussion.

8. Expand Limitations Section: Discuss clinical adoption challenges, ethical concerns, and future improvements.

Response:

Thank you for your valuable suggestion to expand the limitations section by discussing the challenges of clinical adoption, ethical considerations, and potential future improvements. In response to your feedback, we have revised the discussion section and added the following content to address these important aspects:“The clinical adoption of AI-based diagnostic tools like SW-TH01/II faces several challenges. First, the integration of AI into clinical workflows requires extensive training for healthcare providers to interpret AI outputs effectively and to address potential discrepancies between AI and human diagnoses. Second, ethical concerns surrounding AI deployment must be carefully considered, particularly regarding data privacy, algorithm transparency, and accountability in cases of misdiagnosis. Additionally, regulatory approval processes and cost considerations may limit the widespread use of such technologies, especially in resource-limited settings. To address these challenges, future improvements should focus on enhancing the explainability of AI models, ensuring compliance with ethical and legal standards, and reducing the cost of implementation to increase accessibility.”

This addition highlights the key barriers to clinical adoption and the ethical implications of deploying AI-based tools in healthcare. It also outlines actionable directions for future development, such as improving model explainability and ensuring compliance with ethical and legal standards, to facilitate broader adoption. We believe this expanded discussion better contextualizes the limitations of our work and provides a more comprehensive perspective on the challenges and opportunities associated with AI in clinical practice.We appreciate your insightful comments, which have helped us strengthen the manuscript. Thank you again for your constructive feedback.
